# The association between variability, intensity, and persistence of suicidal ideation and prospective suicidal behavior in the systematic treatment enhancement program for bipolar disorder (STEP-BD) study

**DOI:** 10.1186/s40345-022-00263-7

**Published:** 2022-07-01

**Authors:** Bartholt Bloomfield-Clagett, Dede K. Greenstein, Carlos A. Zarate, Elizabeth D. Ballard

**Affiliations:** Experimental Therapeutics & Pathophysiology Branch, National Institute of Mental Health, National Institutes of Health, Building 10, CRC Room 7-5341, 10 Center Drive, MSC 1282, Bethesda, MD 20892 USA

**Keywords:** Suicide, Suicidal ideation, Bipolar disorder, Affective instability

## Abstract

**Background:**

This study sought to examine the association between prospective suicidal behavior and variability, intensity, and persistence of suicidal ideation (SI) in bipolar disorder (BD).

**Methods:**

Data were drawn from the Systematic Treatment Enhancement Program for Bipolar Disorder (STEP-BD), a naturalistic study of 4360 outpatients 15 years or older with BD. In separate models, logistic regressions with suicidal behavior (first attempt or death by suicide) as the outcome variable and SI variability (fluctuating levels of SI over time, measured as ordinal dispersion of SI score), intensity (median SI score over time in study), or persistence (number of visits with reported SI) as the explanatory variables were used to examine the relationship between SI characteristics and odds of future suicidal behavior events.

**Results:**

After adjusting for possible confounders, the odds of prospective suicidal behavior were 1.2 times greater per 10% increase in SI variability. SI persistence was not associated with suicidal behavior. For SI intensity, a median SI score of ‘rare/fleeting’ or ‘several days’ of SI was not associated with suicidal behavior, but the odds of prospective suicidal behavior were nearly five times greater for participants with the highest observed median SI intensity score of ‘nearly every day’.

**Conclusions:**

The findings suggest that, in BD participants, monitoring SI variability may be clinically useful for assessing suicide risk.

**Supplementary Information:**

The online version contains supplementary material available at 10.1186/s40345-022-00263-7.

## Background

Suicidal behavior remains a significant public health burden. In the United States in 2019, over 47,000 individuals died by suicide, and over 312,000 emergency room visits for self-harm were reported (Centers for Disease Control 2021). Accurately assessing suicide risk is critical to mitigating these impacts, and it remains an essential part of current psychiatric practice. However, our ability to accurately predict suicidal behavior in a given individual remains poor, as does our understanding of the inherent mechanisms that lead to this complex, multifactorial behavior. Individuals with bipolar disorder (BD) are at significantly increased risk for suicidal behavior and represent one of many different psychiatric populations for which suicide risk assessment is especially pertinent (Schaffer et al. 2015).

Disease-specific factors are known to influence suicidal behavior despite its transdiagnostic nature, and suicide models in BD have been developed to account for these possible differences. One such model, proposed by Malhi and colleagues, integrates findings gathered from multiple research modalities (ie, genetics and epigenetics, neuroimaging, and clinical research) to describe the suicidal process from ideation to behavior. A tenet of this and other models is the presence of risk factors that exist on a spectrum from proximal (ie, current anxiety) to distal (ie, family history) (Malhi et al. 2018). However, such risk factors may be difficult to detect, unreliably reported, or only indirectly observed.

Few studies have investigated how the parameters of suicidal ideation (SI) itself—including the intensity (average level), duration (sustained levels over time), and variability (fluctuating levels over time) of SI—may predict suicidal behavior. In particular, research in community samples of children (Melhem et al. 2019) and college students (Witte et al. 2005, 2006) as well as in psychiatric populations with suicidal behavior (Bryan et al. 2019; Rizk et al. 2019; Wang et al. 2021) have suggested that SI variability is associated with suicidal behavior, even beyond the effects of SI intensity or SI duration (Rizk et al. 2019). Such fluctuations in SI may be perplexing to clinicians, particularly in patients who have persistent fluctuations in SI but no history of past suicidal behavior, and may even lead them to discount the risk of future suicidal behavior. Thus, it is crucial to investigate patterns of variability in SI and how these patterns may relate to the risk of prospective suicidal behavior. Studies have suggested that different suicidal subgroups (i.e., those with trait-like high and low SI variability) may be associated with different risk factors and clinical characteristics (Bryan et al. 2019; Oquendo et al. 2020). One such study of females with borderline personality disorder and history of suicide attempt concluded that SI variability may be explained by affective instability—broadly defined as fluctuating changes in mood over time—after adjusting for severity of depressive symptoms (Rizk et al. 2019).

Historically, much of the research to elucidate the processes and risk factors associated with suicidal behavior has been conducted in psychiatric subpopulations characterized by specific diagnoses. The Systematic Treatment Enhancement Program for Bipolar Disorder (STEP-BD) study (Sachs et al. 2003) is one such example. Previous research from this naturalistic study of over 4000 individuals with BD described long-term and acute risk factors for suicidal behavior (Antypa et al. 2013; Ballard et al. 2016, 2020; Dennehy et al. 2011; Simon et al. 2007; Stange et al. 2016a, b). The present study used data from the STEP-BD study to investigate the association between suicidal behavior and SI variability, SI intensity, and SI persistence (defined as consistent reporting of SI in the months leading up to a suicidal behavior event) in participants with BD; SI persistence was used because many study participants exhibited suicidal behavior in the months after study entry, and duration could not be directly evaluated. Our study also sought to examine whether the relationship between SI variability and prospective suicidal behavior differed by degree of affective instability. Study hypotheses included: (1) that each of the SI characteristics would be positively associated with suicidal behavior; and (2) that affective instability would modify the relationship between SI variability and suicidal behavior.

## Methods

### Participants

All data for this exploratory analysis were drawn from the STEP-BD study; study characteristics have been described in detail elsewhere (Sachs et al. 2003). Briefly, the 7-year study followed 4360 outpatient participants, ages 15 years or older, with BD of every subtype. The study was conducted across 22 academic medical centers in the United States and was approved by the institutional review board of the participating institutions. All participants provided written informed consent.

The primary study measure was the Clinical Monitoring Form (CMF) (see below). After excluding participants who had no CMF data, our initial dataset included 242 individuals who attempted suicide and eight who died by suicide. Two transgender individuals, both of whom were in the group exhibiting no suicidal behavior, were removed from the analysis due to small sample size. The data were restricted to only include individuals who completed at least three study assessments and who remained in the study for more than 60 days (113 individuals with a suicidal behavior event (SB group) and 3081 individuals without an event (No SB group). The data were further restricted to include only individuals who reported any SI during the study period (which varied by participant). This final dataset comprised 92 participants (64.1% female) in the SB group and 1863 participants (58.9% female) in the No SB group.

### Measures

Data on suicidal behavior, defined as either a suicide attempt or a death by suicide, were collected from the Severe Adverse Events (SAE) and Care Utilization (CU) forms as previously described (Ballard et al. 2020).

The CMF, which was the primary outcome measure in the STEP-BD study, was used to evaluate participants at each outpatient visit with their treating psychiatrist. The CMF measures the severity of DSM-IV mood symptoms (including SI) and other pertinent clinical characteristics within the prior 2-week period. The form has been well-validated and correlates strongly with other mood rating scales such as the Hamilton Depression Rating Scale and the Montgomery-Åsberg Depression Rating Scale (Sachs et al. 2002). Data from the CMF were merged with data from the Affective Disorder Examination (ADE), a semi-structured interview conducted at study entry, which contained the same clinician-administered rating scales found on the CMF. Additionally, information on substance use was drawn from the MINI International Neuropsychiatric Interview (MINI), a brief structured interview for the major Axis I psychiatric disorders also conducted at study entry. Additional details about these forms can be found in the Additional file 1: Methods.

Three explanatory variables were used to examine the relationship between SI characteristics and odds of a future suicidal behavior event: SI variability, SI intensity, and SI persistence. All three were derived from the SI scale of the CMF. Given the ordinal quality of the scale, a statistic for ordinal dispersion was used to assess SI variability over the study period (Blair and Lacy 2000). Briefly, the statistic defines an index of ordinal concentration by taking the sum of the squared difference of the cumulative relative frequency of each level of the scale and the state of maximal dispersion (which is equal to 0.5), and then normalizing by the value for maximal dispersion (given the number of levels of the scale). The difference of this number and one then provides a measure of dispersion of the ordinal variable on a scale from 0 (no dispersion) to 1 (maximal dispersion), where the produced values can be interpreted as the percent of the maximal possible dispersion for the given variable. This approach makes no continuous assumptions about the data and has been shown to produce little bias in estimates with large sample sizes, as is the case with our data (Blair and Lacy 2000). SI intensity was measured as the median SI score per participant over the study period, and SI persistence was measured as the proportion of visits with any reported SI out of the total number of visits over the study period for each participant. Using the ordinal dispersion statistic, measures of affective instability were derived from the DSM-IV mood symptom scales separately for mania and depression (excluding SI), in line with previous approaches (Stange et al. 2016a, b). Severity of depressive symptoms over the study period was measured by summing the absolute value of depressive symptom scores at each visit, again excluding SI, and then averaging these total scores over the study period.

Because past studies have noted a relationship between Personality Disorders Questionnaire (PDQ) score and suicidal behavior in the STEP-BD study (Antypa et al. 2013), this measure was also included (see Additional file 1: Methods).

### Statistical analysis

Summary statistics were calculated for demographics and SI variability, SI intensity, and SI persistence for the SB and No SB groups. Logistic regression was used with suicidal behavior as the outcome variable, and each SI measure of interest (SI variability, SI intensity, or SI persistence) was used as the explanatory variable in separate models to examine the relationship between SI characteristics and likelihood of a future suicidal behavior event.

Covariate selection was conducted to minimize the potential for spurious relationships between predictors of interest and outcome, and Pearson correlation coefficients were calculated with variables of interest and covariates to estimate potential sources of both confounding and collinearity. Based on literature supporting their relationship with either suicidal behavior, SI, or both, the following covariates were included in all models: age at study entry, gender, history of suicide attempt, PDQ score, severity of depressive symptoms, affective instability in depressive and manic symptoms, and alcohol or other substance abuse at study entry (Antypa et al. 2013). Number of total study visits was also included in the models for SI variability, SI intensity, and SI persistence to address possible confounding. To test the hypothesis that affective instability would modify the relationship between SI variability and suicidal behavior, each affective instability measure was interacted with SI variability. As a sensitivity analysis to protect against spurious relationships due to time in study, all of the models were run with only participants who exited the study within two years of study entry; results were similar (see Additional file 1: Table S1).

Multiple imputation was used to account for missingness in the data, which included PDQ score (SB group: 29.8%; No SB group: 21.7%), history of suicide attempt (SB group: 3.2%; No SB group: 1.1%), alcohol abuse (SB group: 6.3%; No SB group: 7.6%), and substance abuse (SB group: 6.4%; No SB group: 7.6%). Regression results from both complete case analyses and multiple imputation analyses were found to be similar. Pooled multiple imputation results are reported below; see Additional file 1: Methods for additional information and Additional file 1: Table S2 for complete case results. All analyses were run using Stata version 16 (StataCorp, College Station, TX, USA).

## Results

Of the 1955 participants included in the analysis, fewer than half were male (41.1% and 35.9% male for the No SB and SB groups, respectively), and most were white (92.0% and 90.2% in the No SB and SB groups, respectively). Mean age at study entry was 40.4 years (SD = 12.2) in the No SB group and 36.4 (SD = 11.6) in the SB group (see Table 1 for additional details).

**Table 1:** Demographic and clinical characteristics

	No suicidal behavior (n = 1863)	Suicidal behavior (*n* = 92)	Missing	
			No suicidal behavior	Suicidal behavior
Age at study entry	40.43 ± 12.23	36.39 ± 11.63		
Gender				
Male	765 (41.1)	33 (35.9)		
Female	1098 (58.9)	59 (64.1)		
Race			1 (< 0.1)	
White	1714 (92.0)	83 (90.2)		
Non-white	148 (8.0)	9 (9.8)		
Hispanic ethnicity	92 (4.9)	1 (1.1)	1 (< 0.1)	
SI variability				
SI score dispersion × 10^a^	2.87 ± 1.61	3.63 ± 1.56		
SI intensity				
SI median score				
No SI	1482 (79.5)	59 (64.1)		
Rare/fleeting	167 (9.0)	12 (13.0)		
Several days	179 (9.6)	11 (12.0)		
Nearly every day	35 (1.9)	10 (10.9)		
SI persistence				
Proportion of visits with SI × 10^a^	3.07 ± 2.34	4.07 ± 2.55		
Affective instability				
Depressive symptoms	0.38 ± 0.11	0.40 ± 0.09		
Manic symptoms	0.22 ± 0.12	0.24 ± 0.13		
Depression severity	3.91 ± 1.94	4.76 ± 1.99		
Hx of suicide attempt	716 (38.4)	62 (67.4)	59 (3.2)	1 (1.1)
PDQ score	37.03 ± 16.03	40.83 ± 14.66	555 (29.8)	20 (21.7)
Alcohol abuse	197 (10.6)	19 (20.6)	117 (6.3)	7 (7.6)
Substance abuse	125 (6.7)	15 (16.3)	120 (6.4)	7 (7.6)

*SI* suicidal ideation, *PDQ* Personality Disorders Questionnaire

^a^A one unit change corresponds to a 10% change in variable (SI score dispersion or proportion of visits with SI)

**Fig. 1:**
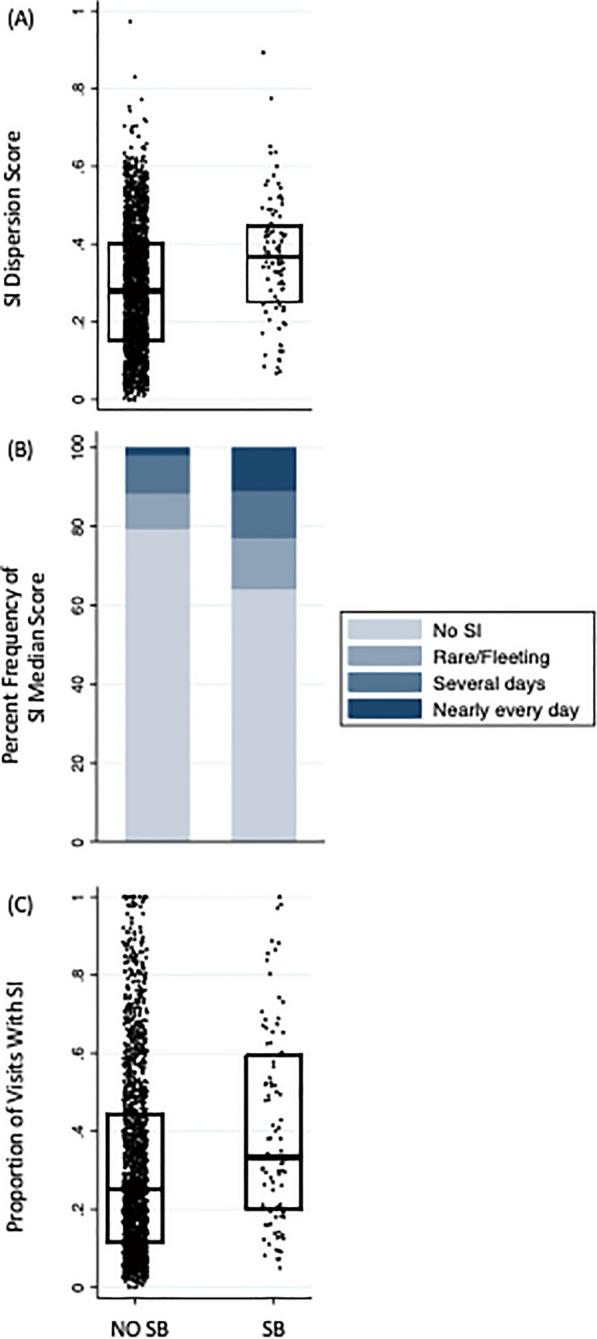
Distribution of suicidal ideation (SI) variability, intensity, and persistence by outcome group [suicidal behavior (SB) versus no suicidal behavior (No SB)]. **A** SI variability: box plot with interquartile range of SI dispersion score; **B** SI intensity: percent frequency of SI median score (no SI, rare/fleeting, several days, nearly every day); **C** SI persistence: box plot with interquartile range of proportion of visits with SI

Participants demonstrated a relatively broad range of SI variability [No SB group: 2.87 ± 1.61 (range = 0–9.50); SB group: 3.63 ± 1.56 (range = 0.64–8.89)] as well as SI persistence [No SB group: 3.07 ± 2.34 (range = 0.17 to 10.00); SB group: 4.07 ± 2.55 (range = 0.42 to 10.00)]. SI intensity scores, as measured by median CMF SI score, were clustered near lower scores, suggesting the absence of SI (percent of individuals with median score of no SI: 79.5% in the No SB group and 64.1% in the SB group) (Table 1 and Fig. 1).

Regarding model selection, number of study visits and time in study were not included in the same models because these variables were highly collinear (rs = 0.78) (Table 2) (see Additional file 1: Results for further details), and further adjusting for time in study did not substantially change the results after already adjusting for number of study visits. Affective instability did not modify the relationship between SI variability and prospective suicidal behavior (SI variability × depressive symptom instability: odds ratio (OR) = 0.73, p = 0.67, SI variability × manic symptom instability: OR = 1.19, p = 0.73). Consequently, these interaction terms were not included in the final model for SI variability.

**Table 2:** Spearman correlations for predictor variables (*n* = 1955)

	1	2	3	4	5	6	7	8	9	10	11	12	13	14
SI score dispersion	1.00													
SI median score	0.45	1.00												
Proportion of visits with SI	0.81	0.71	1.00											
Age at study entry	0.00	0.04	0.02	1.00										
Gender	0.01	− 0.06	− 0.02	− 0.09	1.00									
History of suicide attempt	0.16	0.08	0.15	− 0.01	0.12	1.00								
PDQ score	0.18	0.17	0.21	− 0.12	− 0.02	0.16	1.00							
Depression severity	0.47	0.39	0.49	0.05	0.07	0.14	0.35	1.00						
Depression symptom instability	0.32	0.07	0.18	0.00	0.09	0.12	0.23	0.59	1.00					
Manic symptom instability	0.18	0.10	0.12	− 0.09	0.10	0.12	0.30	0.33	0.46	1.00				
Alcohol abuse	0.02	− 0.03	− 0.02	− 0.15	− 0.12	0.03	0.12	0.01	0.05	0.03	1.00			
Substance abuse	− 0.01	− 0.01	0.00	− 0.13	− 0.09	0.03	0.12	− 0.02	0.03	0.04	0.35	1.00		
Total number of visits	− 0.14	− 0.16	− 0.24	0.10	0.03	− 0.05	− 0.15	− 0.10	0.20	0.04	− 0.07	− 0.07	1.00	
Days in study	− 0.21	− 0.23	− 0.32	0.11	0.00	− 0.06	− 0.24	− 0.24	0.08	− 0.01	− 0.08	− 0.08	0.78	1.00

*SI* suicidal ideation, *PDQ* Personality Disorders Questionnaire

After adjusting for possible confounders, logistic regressions indicated that the odds of prospective suicidal behavior were 1.2 times greater per 10% increase in SI variability (OR = 1.21, p = 0.01). In contrast, SI persistence (OR = 1.10, p = 0.05) was not associated with suicidal behavior. For SI intensity, a median SI score of ‘rare/fleeting’ (OR = 1.54, p = 0.21) or ‘several days’ of SI (OR = 1.32, p = 0.45) was not associated with suicidal behavior compared with the reference group of no SI, but the odds of prospective suicidal behavior was nearly five times greater for participants with the highest observed median SI score of ‘nearly every day’ (OR = 4.81, p < 0.1) (Table 3).

**Table 3:** Odds ratios for predictors of prospective suicidal behavior (*n* = 1955)

	OR	95% CI	*P*
SI variability model			
SI score dispersion × 10^a^	1.21	1.05–1.40	0.01*
Age at study entry	0.98	0.96–1.00	0.02*
Gender	1.08	0.68–1.72	0.74
History of suicide attempt	2.77	1.73–4.45	< 0.01*
PDQ score	1.00	0.98–1.01	0.63
Depression severity	1.13	0.99–1.28	0.05
Depression symptom instability	1.02	0.08–13.17	0.99
Manic symptom instability	0.81	0.11–6.00	0.84
Alcohol abuse	1.52	0.83–2.79	0.17
Substance abuse	2.04	1.04–4.03	0.04*
Total number of visits	0.98	0.96–1.00	0.04*
SI intensity model			
SI median score			
No SI	*Reference*		
Rare/fleeting	1.54	0.79–3.03	0.21
Several days	1.32	0.64–2.76	0.45
Nearly every day	4.81	1.99–11.60	< 0.01*
Age at study entry	0.97	0.96–0.99	0.01*
Gender	1.11	0.70–1.79	0.65
History of suicide attempt	2.79	1.73–4.50	< 0.01*
PDQ score	1.00	0.98–1.01	0.58
Depression severity	1.09	0.95–1.25	0.21
Depression symptom instability	3.34	0.25–45.28	0.37
Manic symptom instability	0.70	0.09–5.35	0.73
Alcohol abuse	1.65	0.90–3.05	0.10
Substance abuse	1.84	0.93–3.64	0.08
Total number of visits	0.98	0.96–1.00	0.03*
SI persistence model			
Proportion of visits with SI × 10^a^	1.10	1.00–1.22	0.05
Age at study entry	0.98	0.96–1.00	0.02*
Gender	1.09	0.70–1.73	0.70
History of suicide attempt	2.79	1.75–4.43	< 0.01*
PDQ score	0.99	0.98–1.01	0.58
Depression severity	1.11	0.98–1.25	0.10
Depression symptom instability	2.53	0.26–24.5	0.42
Manic symptom instability	0.81	0.11–5.95	0.84
Alcohol abuse	1.64	0.89–3.02	0.11
Substance abuse	1.86	0.96–360	0.07
Total number of visits	0.98	0.96–0.99	0.04*

*SI* suicidal ideation, *PDQ* Personality Disorders Questionnaire

^a^A one unit change corresponds to a 10% change in variable (SI score dispersion or proportion of visits with SI); * *p* < .05

## Discussion

The findings did not fully support the first hypothesis that each SI characteristic would be positively associated with suicidal behavior. Rather, SI variability was found to be significantly associated with prospective suicidal behavior for BD participants, and SI persistence was not. In addition, only the highest level of SI intensity (corresponding to a median SI score of ‘nearly every day’) was associated with prospective suicidal behavior. The results also did not support the second hypothesis that affective instability would modify the relationship between SI variability and suicidal behavior. Taken together, these findings suggest that monitoring SI variability may be a useful clinical tool for assessing suicide risk.

These findings are consistent with previous research suggesting that SI variability may explain prospective suicidal behavior (Wang et al. 2021; Witte et al. 2005). It should be noted that the relatively small magnitude of this result is not unexpected and in line with estimates of other risk factors, such as marital status, reported in previous research studies (Pescolido et al. 2020). Notably, the SI intensity level of ‘nearly every day’ was relatively rare in our suicidal sample (2.3%). High SI variability may indicate that a more in-depth suicide risk assessment is needed, especially among those without high SI intensity, which would appear to comprise most patients with SI.

Interestingly, affective instability—broadly defined as fluctuating changes in mood over time—did not modify the relationship between SI variability and prospective suicidal behavior, suggesting that this relationship may occur independently of overall mood changes. Prior research on this topic has produced mixed results. While some studies reported an association between affective instability and history of suicidal behavior across a range of psychiatric populations (Palmier-Claus et al. 2012) as well as an association between severity of BD and history of suicidal behavior (Fagiolini et al. 2004; Oquendo et al. 2000), another study of BD participants found no evidence of a relationship between affective instability and history of suicidal behavior (Parmentier et al. 2012). One possible explanation is that there may be little association between affective instability and suicidal behavior after accounting for severity of depressive symptoms if individuals with higher affective instability spend less time in a state of severe mood symptoms, where risk of suicidal behavior may be highest.

The absence of a finding of effect modification for affective instability may also be due to a number of study-specific reasons. First, the models used assessed effect modification on the multiplicative scale and not on the additive scale. Second, how researchers conceptualize and measure affective instability may differ between studies and study populations. Our finding contrasts with one observed in patients with borderline personality disorder (Rizk et al. 2019), but the constructs of affective instability may differ between these two populations (Reich et al. 2012) and were measured differently in the two studies. Third, it is possible that our separate measures of depressive and manic symptom instability did not capture the full spectrum or temporal nature of these dynamic symptoms. While our ability to capture this construct was in line with previous research efforts (Stange et al. 2016a, b), it was nevertheless limited by the information collected on the CMF; newer methods of data collection, such as ecological momentary assessment (EMA), may address these issues (Kleiman et al. 2017).

Given that most suicidal behavior events took place early in a participant’s time course, this study could not assess the association between duration of SI and suicidal behavior. Instead, the measure of proportion of visits with reported SI primarily provides insight into the degree of SI persistence in the months leading up to a suicidal behavior event compared with those without an event. When restricting the analysis to participants who exited the study within the first 2 years of entry, an association remained between SI variability and suicidal behavior, but there was no clear evidence of an association between SI persistence and suicidal behavior.

Strengths of the present study include the large sample size, the longitudinal nature of the data, and the standardized diagnosis, assessment, and treatment of the BD participants. The large sample size is particularly important given the lower incidence of suicidal behaviors. This analysis also expands on previous work in this area by: (1) extending research on SI variability to individuals with BD; (2) investigating the association between prospective suicidal behavior rather than history of suicide attempt or future SI; and (3) examining these relationships in a large, community-based sample.

This analysis also has several limitations. First, because this was an exploratory analysis, it did not adjust for multiple testing, and statistically significant results should therefore be interpreted with caution. Second, the CMF was collected at outpatient follow-up visits and depended on both the clinician determination of need as well as participant behavior, which may have had a confounding effect on study results. For example, patients with lesser degrees of SI may not have been seen as frequently, or those with greater degrees of SI may have been more likely to be non-adherent to outpatient visits or more likely to be hospitalized, reducing their number of outpatient visits. The analysis attempted to partially account for these confounding effects by including the number of visits and time spent in the study into the models, and by restricting the analyses to the first two years from study entry; this approach, however, may not have fully accounted for these effects and, again, the results should be interpreted with caution. Third, while the study psychiatrists were trained on both the CMF and best practices for care of patients with BD, they may have behaved differently regarding the degree to which they adhered to the CMF interview and reporting form. Because the de-identified dataset does not include data at the provider level, the analysis could not account for these possible effects. Fourth, the STEP-BD study was conducted at academic medical centers where participants were responsible for covering the costs of their treatment; there was substantial drop-out over the course of the STEP-BD study and, consequently, the results may not be generalizable to other populations, such as patients seen in community clinics where participants may have been more severely ill or had different insurance coverage. Fifth, because SI severity—defined as maximum SI score—correlated highly with SI variability, it was not possible to distinguish between the effects of SI severity and SI variability in this dataset. Sixth, it is possible that participants in the No SB group went on to experience suicidal behavior after exiting the study, potentially limiting the generalizability of the findings. Finally, while the models showed good fit, using them to directly categorize risk of future suicidal behavior would be substantially limited because they did not predict the full range of values for predicted probability (about 0 to 0.6). This is likely due to the unbalanced nature of the data, given the overall low rates of suicidal behavior. Furthermore, using the models for prediction would require validation in additional datasets.

## Conclusion

SI variability may help explain risk of suicidal behavior as well as SI and could be considered in clinical contexts in addition to other routinely-assessed, common risk factors, particularly among individuals with lower SI intensity. Future studies are warranted to replicate the above analysis in novel datasets in order to assess the reliability of the findings, to extend this research to other transdiagnostic patient populations, and to investigate the possible neurobiological underpinnings of a relationship between SI variability and the urge to act on suicidal thoughts.

## Supplementary Information

**Additional file 1.** Supplementary methods and results

## Data Availability

The data that support the findings of this study are openly available in the NIMH Data Archive (https://nda.nih.gov/study.html?id=437), DOI: 10.15154/1338252, ID: 2147.

## Abbreviations

ADE: Affective disorder examination
BD: Bipolar disorder
CMF: Clinical monitoring form
CU: Care utilization
EMA: Ecological momentary assessment
MINI: MINI international neuropsychiatric interview
PDQ: Personality disorders questionnaire
OR: Odds ratio
SAE: Severe adverse event
SB: Suicidal behavior
SI: Suicidal ideation
STEP-BD: Systematic Treatment Enhancement Program for Bipolar Disorder
