# Supplementary material for: The association between variability, intensity, and persistence of suicidal ideation and prospective suicidal behavior in the systematic treatment enhancement program for bipolar disorder (STEP-BD) study
Source: Int J Bipolar Disord. 2022 Jul 1;10:17. doi: 10.1186/s40345-022-00263-7 (PMC9247121; doi:10.1186/s40345-022-00263-7)
Supplement: Supplementary file 1 — Additional file 1. Supplementary methods and results [file 40345_2022_263_MOESM1_ESM.docx]

*Bloomfield-Clagett et al, “The Association Between Variability, Intensity, and Persistence of Suicidal Ideation and Prospective Suicidal Behavior in the Systematic Treatment Enhancement Program*

*for Bipolar Disorder (STEP-BD) Study”*

**Additional file 1**

**Methods**

*Severe Adverse Events (SAE) and Care Utilization (CU) Forms*

SAE forms, which recorded the dates of suicide attempts as well as deaths by suicide, were reviewed by an independent safety committee. CU forms, which asked participants whether they had attempted suicide in the past three months, were collected quarterly during the first year of study participation and semi-annually thereafter. Data from both forms were collapsed for use in this analysis. In instances where the same suicidal behavior event was likely to be reported on both forms, the information from the SAE was retained. Given the prospective nature of the analysis, data were discarded if an individual noted a suicidal behavior event on the CU form in the first three months of study participation but did not report the absence of a recent suicide event in prior or baseline data. Additionally, data were censored at the time of a first suicidal behavior event (if the event was reported on the SAE form) or at the time of study assessment where reported (if the event was reported on the CU form).

*Clinical Monitoring form (CMF)*

On the CMF form, DSM-IV mood symptoms are reported on scales ranging from -2 to 0, 0 to +2, or -2 to +2, depending on the nature of the mood symptom. The scale for SI is as follows: 0 (none), +.25 (rare/fleeting), +.5 (several days, fleeting; or several days, persistent periods), +1 (nearly every day, persistent most of the day; nearly every day, any period of persistent; or several days, brief and active SI), +1.5 (greater than one persistent period and active SI), and +2 (any frequency of active SI with urges to self-harm).

*Personality Disorder Questionnaire (PDQ)*

The PDQ is a 99-item self-report measure used to identify the primary features of a personality disorder as defined by DSM-IV criteria. Reponses are indicated as true or false. The total sum of the scale is used to measure the likelihood of the presence of a personality disorder.

*Affective Instability*

A value of ordinal dispersion was computed for each DSM-IV mood symptom for each participant across time. The mean of the dispersion values across all depressive symptoms, excluding SI, was then computed to establish a measure of affective instability in depressive symptoms for each participant. Affective instability in manic symptoms for each participant was similarly derived.

*Model Fit*

Likelihood ratio tests of nested models were used to assess whether covariates contributed significantly to the prediction of suicidal behavior. Model fit was assessed using residuals plots, Hosmer-Lemeshow tests, and calibration and discrimination metrics.

*Multiple Imputation*

Multiple imputation by chained equations with 50 imputations was used to impute missing PDQ, suicide attempt history, alcohol abuse, and substance abuse data. A binomial distribution was assumed for history of suicide attempt, alcohol abuse, and substance abuse, while multivariate normal distribution was assumed for PDQ score truncated by the possible score values (0 to 99). There was no other missingness in the data. All variables from each regression model were used in the imputation model. Stata MI impute and MI estimate (StataCorp, College Station, TX, USA), which use combination rules to produce estimates across imputations, were used to perform the multiple imputation analyses (Rubin 1987).

**Results**

*Model Selection*

Compared to models that only contained age, history of suicide attempt, alcohol abuse, PDQ score, and the SI variable of interest, including total number of study visits significantly improved fit for the SI variability (χ2=11.38, p<0.01) and SI intensity models (χ2=10.33, p<0.01). Time (days since entry to the study) improved fit of the SI persistence model (χ2=38.47, p<0.01). Severity of depressive symptoms, affective instability, substance use, and proportion of study visits where recent headaches were reported did not improve fit for any of the models (SI variability; χ2=7.83, p=0.17; SI intensity: χ2=5.68, p=0.34; SI persistence: χ2=5.47, p=0.36). Hosmer-Lemeshow tests, which were used to assess goodness of fit, showed adequate fit for the SI variability (χ2=3.91, p=0.86), SI intensity (χ2=6.14, p=0.63), and SI persistence models (χ2=2.40, p=0.97).

**References**

Rubin DB. Multiple imputation for nonresponse in surveys. New York, New York: John Wiley & Sons; 1987.

| Table S1. Odds Ratios of Predictors of Prospective Suicidal Behavior for Participants Exiting the STEP-BD Study within 2 Years (n=1,217) | | | | | |  |
| --- | --- | --- | --- | --- | --- | --- |
|  |  |  |  |  |  |  |
|  |  |  | OR | 95% CI | *p* |  |
| SI Variability Model | | |  |  |  |  |
|  | SI Score Dispersion x 10^a^ | | 1.20 | 1.03-1.41 | **.02** |  |
|  | Age at Study Entry | | 0.98 | 0.95-1.00 | .**02** |  |
|  | Gender | | 1.01 | 0.61-1.67 | .98 |  |
|  | History of Suicide Attempt | | 2.76 | 1.67-4.59 | **<.01** |  |
|  | PDQ Score | | 0.99 | 0.98-1.01 | .58 |  |
|  | Depression Severity | | 1.11 | 0.98-1.27 | .11 |  |
|  | Depressive Symptom Instability | | 1.90 | 0.12-29.3 | .65 |  |
|  | Manic Symptom Instability | | 0.41 | 0.05-3.55 | .41 |  |
|  | Alcohol Abuse | | 1.66 | 0.89-3.12 | .11 |  |
|  | Substance Abuse | | 2.07 | 1.01-4.26 | .05 |  |
|  | Total Number of Visits | | 1.00 | 0.97-1.03 | .81 |  |
|  |  |  |  |  |  |  |
| SI Intensity Model | | |  |  |  |  |
|  | SI Median Score | |  |  |  |  |
|  |  | No SI | *Reference* |  |  |  |
|  |  | Rare/Fleeting | 1.65 | 0.83-3.29 | .16 |  |
|  |  | Several days | 1.10 | 0.50-2.45 | .81 |  |
|  |  | Nearly every day | 4.62 | 1.79-11.97 | **<.01** |  |
|  | Age at Study Entry | | 0.97 | 0.95-0.99 | **.01** |  |
|  | Gender | | 1.04 | 0.62-1.73 | .88 |  |
|  | History of Suicide Attempt | | 2.76 | 1.66-4.59 | **<.01** |  |
|  | PDQ Score | | 0.99 | 0.98-1.01 | .50 |  |
|  | Depression Severity | | 1.08 | 0.93-1.24 | .33 |  |
|  | Depressive Symptom Instability | | 5.40 | 0.33-88.69 | .24 |  |
|  | Manic Symptom Instability | | 0.35 | 0.04-3.18 | .35 |  |
|  | Alcohol Abuse | | 1.82 | 0.96-3.43 | .07 |  |
|  | Substance Abuse | | 1.87 | 0.91-3.84 | .09 |  |
|  | Total Number of Visits | | 1.00 | 0.97-1.03 | .83 |  |
|  |  |  |  |  |  |  |
| SI Persistence Model | | |  |  |  |  |
|  | Proportion of Visits with SI x 10^a^ | | 1.05 | 0.93-1.19 | .41 |  |
|  | Age at Study Entry | | 0.98 | 0.95-1.00 | .11 |  |
|  | Gender | | 0.99 | 0.54-1.80 | .96 |  |
|  | History of Suicide Attempt | | 2.51 | 1.41-4.46 | **<.01** |  |
|  | PDQ Score | | 1.00 | 0.98-1.01 | .65 |  |
|  | Depression Severity | | 1.08 | 0.91-1.29 | .39 |  |
|  | Depressive Symptom Instability | | 3.01 | 0.13-71.47 | .50 |  |
|  | Manic Symptom Instability | | 0.45 | 0.03-6.10 | .55 |  |
|  | Alcohol Abuse | | 1.59 | 0.76-3.33 | .22 |  |
|  | Substance Abuse | | 1.99 | 0.90-4.43 | .09 |  |
|  | Total Number of Visits | | 0.97 | 0.96-1.03 | .83 |  |

^a^a one unit change corresponds to a 10% change in variable (SI score dispersion or proportion of visits with SI)

SI: suicidal ideation; PDQ: Personality Disorders Questionnaire

| Table S2. Odds Ratios of Predictors of Prospective Suicidal Behavior (Complete Case Analysis) | | | | | | | | |  |
| --- | --- | --- | --- | --- | --- | --- | --- | --- | --- |
|  |  |  | All Participants (*n*=1,287) | | |  | Participants Exiting Within 2 Years (*n*=721) | | |
|  |  |  | OR | 95% CI | *p* |  | OR | 95% CI | *p* |
| SI Variability Model | | |  |  |  |  |  |  |  |
|  | SI Score Dispersion x 10^a^ | | 1.23 | 1.04-1.46 | **.02** |  | 1.24 | 1.03-1.49 | **.02** |
|  | Age at Study Entry | | 0.98 | 0.96-1.00 | .12 |  | 0.98 | 0.96-1.01 | .13 |
|  | Gender | | 1.21 | 0.69-2.12 | .51 |  | 1.02 | 0.56-1.87 | .94 |
|  | History of Suicide Attempt | | 2.44 | 1.42-4.17 | **<.01** |  | 2.44 | 1.37-4.33 | **<.01** |
|  | PDQ Score | | 1.00 | 0.98-1.02 | .78 |  | 1.00 | 0.98-1.02 | .69 |
|  | Depression Severity | | 1.11 | 0.96-1.29 | .17 |  | 1.08 | 0.92-1.26 | .36 |
|  | Depressive Symptom Instability | | 1.04 | 0.05-21.26 | .98 |  | 1.26 | 0.05-30.76 | .89 |
|  | Manic Symptom Instability | | 0.57 | 0.05-6.50 | .65 |  | 0.43 | 0.03-5.75 | .52 |
|  | Alcohol Abuse | | 1.28 | 0.63-2.60 | .49 |  | 1.49 | 0.71-3.11 | .29 |
|  | Substance Abuse | | 2.13 | 0.99-4.60 | .05 |  | 2.25 | 1.00-5.05 | .05 |
|  | Total Number of Visits | | 0.04 | 0.94-0.99 | **<.01** |  | 1.24 | 0.96-1.04 | .95 |
|  |  |  |  |  |  |  |  |  |  |
| SI Intensity Model | | |  |  |  |  |  |  |  |
|  | SI Median Score | |  |  |  |  |  |  |  |
|  |  | No SI | *Reference* | |  |  | *Reference* |  |  |
|  |  | Rare/Fleeting | 1.96 | 0.91-4.23 | .09 |  | 2.09 | 0.94-4.62 | .07 |
|  |  | Several days | 1.10 | 0.43-2.79 | .84 |  | 0.98 | 0.36-2.64 | .97 |
|  |  | Nearly every day | 4.29 | 1.43-12.86 | **.01** |  | 4.10 | 1.20-14.01 | **.03** |
|  | Age at Study Entry | | 0.98 | 0.96-1.00 | .09 |  | 0.98 | 0.95-1.00 | .08 |
|  | Gender | | 1.22 | 0.69-2.16 | .48 |  | 1.03 | 0.56-1.89 | .92 |
|  | History of Suicide Attempt | | 2.45 | 1.42-4.22 | **<.01** |  | 2.44 | 1.36-4.39 | **<.01** |
|  | PDQ Score | | 1.00 | 0.98-1.02 | .73 |  | 1.00 | 0.98-1.01 | .62 |
|  | Depression Severity | | 1.08 | 0.91-1.28 | .41 |  | 1.05 | 0.87-1.25 | .63 |
|  | Depressive Symptom Instability | | 4.43 | 0.20-97.17 | .35 |  | 4.73 | 0.18-127.51 | .36 |
|  | Manic Symptom Instability | | 0.43 | 0.04-5.13 | .50 |  | 0.33 | 0.02-4.70 | .41 |
|  | Alcohol Abuse | | 1.43 | 0.70-2.93 | .33 |  | 1.67 | 0.79-3.52 | .18 |
|  | Substance Abuse | | 1.87 | 0.86-4.07 | .11 |  | 1.96 | 0.87-4.39 | .10 |
|  | Total Number of Visits | | 0.96 | 0.94-0.99 | **<.01** |  | 1.00 | 0.96-1.03 | .91 |
|  |  |  |  |  |  |  |  |  |  |
| SI Persistence Model | | |  |  |  |  |  |  |  |
|  | Proportion of Visits with SI x 10 ^a^ | | 1.08 | 0.96-1.22 | .18 |  | 1.05 | 0.93-1.19 | .41 |
|  | Age at Study Entry | | 0.98 | 0.96-1.00 | .11 |  | 0.98 | 0.95-1.00 | .11 |
|  | Gender | | 1.19 | 0.68-2.09 | .54 |  | 0.99 | 0.54-1.80 | .96 |
|  | History of Suicide Attempt | | 2.46 | 1.44-4.21 | **<.01** |  | 2.51 | 1.41-4.46 | **<.01** |
|  | PDQ Score | | 1.00 | 0.98-1.02 | .72 |  | 1.00 | 0.98-1.01 | .65 |
|  | Depression Severity | | 1.10 | 0.93-1.30 | .29 |  | 1.08 | 0.91-1.29 | .39 |
|  | Depressive Symptom Instability | | 2.96 | 0.15-59.04 | .48 |  | 3.01 | 0.13-71.47 | .50 |
|  | Manic Symptom Instability | | 0.57 | 0.05-6.50 | .65 |  | 0.45 | 0.03-6.10 | .55 |
|  | Alcohol Abuse | | 1.42 | 0.70-2.89 | .34 |  | 1.59 | 0.76-3.33 | .22 |
|  | Substance Abuse | | 1.90 | 0.88-4.07 | .10 |  | 2.00 | 0.90-4.43 | .09 |
|  | Total Number of Visits | | 0.96 | 0.940.99 | **.01** |  | 1.00 | 0.96-1.03 | .83 |

^a^a one unit change corresponds to a 10% change in variable (SI score dispersion or proportion of visits with SI)
